# Production of Corn Protein Hydrolysate with Glutamine-Rich Peptides and Its Antagonistic Function in Ulcerative Colitis In Vivo

**DOI:** 10.3390/foods11213359

**Published:** 2022-10-25

**Authors:** Yan Jing, Xiaolan Liu, Jinyu Wang, Yongqiang Ma, Xiqun Zheng

**Affiliations:** 1 College of Food Engineering, Harbin University of Commerce, Harbin 150076, China; 2 Key Laboratory of Corn Deep Processing Theory and Technology of Heilongjiang Province, College of Food and Bioengineering, Qiqihar University, Qiqihar 161006, China; 3 College of Food, Heilongjiang Bayi Agricultural University, Daqing 163319, China

**Keywords:** corn gluten meal, glutamine, ulcerative colitis

## Abstract

Ulcerative colitis is a typical chronic inflammatory disease of the gastrointestinal tract, which has become a serious hazard to human health. The purpose of the present study was to evaluate the antagonistic effect of corn protein hydrolysate with glutamine-rich peptides on ulcerative colitis. The sequential hydrolysis of corn gluten meal by Alcalase and Protamex was conducted to prepare the hydrolysate, and then the mouse ulcerative colitis model induced by dextran sulfate sodium was applied to evaluate its biological activities. The results indicated that the hydrolysate significantly improved weight loss (*p* < 0.05), reduced the colonic shortening and the disease activity index, diminished the infiltration of inflammatory cells in the colonic tissue, and reduced the permeability of the colonic mucosa in mice. In addition, the hydrolysate decreased the contents of pro-inflammatory factors IL-1β, IL-6, and TNF-α, increased the anti-inflammatory factor IL-10 and oxidative stress markers GSH-Px and SOD in the animal tests. Moreover, the hydrolysate also regulated the abundance and diversity of the intestinal microbiota, improved the microbiota structure, and increased the content of beneficial bacteria including *Lactobacillus* and *Pediococcus*. These results indicated that the hydrolysate might be used as an alternative natural product for the prevention of ulcerative colitis and could be further developed into a functional food.

## 1. Introduction

Inflammatory bowel disease (IBD) mainly includes ulcerative colitis (UC) and Crohn’s disease (CD), and it is a complex disease of the digestive system characterized by repeated chronic intestinal inflammation, intestinal epithelial dysfunction, and mucosal tissue damage in the gastrointestinal tract [1]. Epidemiology shows that in recent years, the prevalence of UC has been on the rise worldwide [2,3]. Multiple causes of UC have been identified, and its consequences usually include the impaired structure and function of the intestinal mucosa [4]. At present, the main drugs for the treatment of UC include salicylazosulfapyridine, immunosuppressants, and an anti-tumor necrosis factor-α monoclonal antibody. Unfortunately, the long-term use of these drugs can cause serious side effects and are expensive, which severely limits their use [5,6]. Hence, safer and more efficient naturally active substances for the treatment of UC are urgently needed.

Glutamine (Gln) is one of the most abundant amino acids in the human body and has many physiological functions, such as enhancing the immune capacity of the body and protecting the intestinal barrier function [7,8]. However, the application of Gln is limited due to its unstable monomer, easy decomposition, and low solubility. A few studies have found that glutamine peptide not only retains a variety of physiological functions and nutrition of Gln, but also has good water solubility and stability, which is a stable substitute for Gln. Researchers have also found that glutamine peptide has antioxidant activity, promotes protein synthesis, and protects human intestinal health function. Under stress conditions such as fatigue, intense exercise, and disease, glutamine peptides can be rapidly decomposed into glutamine to meet the needs of the body. For example, Juan Tian et al. reported that alanine–glutamine could attenuate soya saponin-induced growth inhibition and intestinal injury in zebrafish [9]. Bie Tan et al. found that alanine–glutamine could replace glutamine as a source of energy and protein in the gastrointestinal tract [10]. Therefore, the development of bioactive peptides rich in Gln can effectively solve the problem of the limited application of Gln. 

Corn is one of the three major cereals in the world, and China’s yield of corn reached 272.55 million tons in 2021. Nearly one-sixth of corn is used to produce corn starch using the wet milling process. It generates a large amount of corn gluten meal (CGM) as a main co-product, which contains about 67–72% (*w/w*) protein [11,12]. These proteins mainly include zein and gluten; the content of Gln is about one-third of the total amino acids in gluten [13,14] and is an excellent raw material for preparing glutamine active peptides. Therefore, corn protein hydrolysate with glutamine-rich peptides (APH) was first prepared from the hydrolysis of CGM by proteases in the present study, and then its antagonistic effect on DSS-induced UC in vivo was systematically evaluated.

## 2. Materials and Methods

### 2.1. Materials

CGM was purchased from Longjiang Fufeng Biotechnology Co., Ltd. (Qiqihar, China). The proteases Alcalase and Protamex were purchased from Novo Nordisk (Bagsvaerd, Denmark). DSS (36–50 kDa) was purchased from Macklin (Shanghai, China). All of the other reagents and chemicals were of analytical grade.

### 2.2. Preparation of CGM Hydrolysates

The CGM was pretreated using the method described in our previous report [15,16]. Pretreated CGM (20 g) was added to 200 mL of distilled water, and then Alcalase and Protamex were added successively for hydrolysis. The optimum hydrolysis conditions for Alcalase were a temperature of 64 °C, hydrolysis time of 2.5 h, amount of enzyme of 1300 U/g, and a pH of 8.0. The optimum hydrolysis conditions for Protamex were a temperature of 50 °C, hydrolysis time of 2.5 h, amount of enzyme of 200 U/g, and a pH of 7.0. For the sequential hydrolysis of the two proteases, first of all, the CGM was hydrolyzed with one protease (Alcalase or Protamex), and then the second protease was added after adjusting the reaction conditions. After the reaction, the hydrolysates were centrifuged at 4500 rpm for 15 min to collect the supernatant and were lyophilized.

### 2.3. Determination of Gln Content in Hydrolysates

The Gln content of the corn protein hydrolysate was determined by Katharina’s method [17]. Briefly, 500 μL corn protein hydrolysate (100 mg/mL) was transferred into an ampoule (Hawk, Harbin, China), and then 2 mL bis-1,1-trifluoroacetoxy-iodobenz-ene (BTI) acetonitrile-aqueous solution (10 mg/mL) and 500 μL pyridine (50 μmol/mL) were added into the ampoule. The reaction was carried out at 50 °C for 2 h to protect the Gln in the hydrolysate, and the other hydrolysate sample was not subjected to BTI protection reaction. Then, 5 mL HCl (6 mol/L) was added to the two ampoules for acid hydrolysis, and the ampoules were sealed in a vacuum and hydrolyzed at 110 °C for 24 h. After cooling, the pH of the hydrolysate was adjusted to 6–8 with NaOH, and the glutamic acid content was determined using a glutamate biosensor analyzer; the difference in the glutamic acid between the two samples was the content of Gln. 

### 2.4. Animals and Experimental Design

Male Kunming mice (6–8 weeks) were provided by the Laboratory Animal Technology of Changchun Yisi (Changchun, Jilin, China, Permission No. SCXK (JI) 2020-0002). The animal study protocol was approved on 25 March 2021 by the Animal Ethics Committee of College of Food and Bioengineering, Qiqihar University (Approval No. 2021-003). The care and treatment of the mice were in accordance with international guidelines for laboratory animals (Association for Assessment and Accreditation of Laboratory Animal Care, AAALAC). Before the experiment, all animals were fed and watered freely for one week, and the room temperature was maintained at 23 ± 3 °C. Then, the mice were randomly divided into five groups (*n* = 10). All mice were fed a normal diet during the experiment, and dextran sulfate sodium (DSS) was used as an inducer to establish the colitis model. In GroupⅠ (control group), the mice received drinking water; in GroupⅡ (DSS model group), the mice received drinking water for the first 7 days, and drinking water containing 3% DSS for the next 7 days; GroupⅢ (APH low-dose group) received 100 mg kg^−1^ APH + 3% DSS; GroupⅣ (APH medium-dose group) received 300 mg kg^−1^ APH + 3% DSS; and GroupⅤ (APH high-dose group) received 500 mg kg^−1^ APH + 3% DSS. During the experiment, the APH group mice were given the corresponding APH dose by gavage daily and treated with drinking water containing 3% DSS from days 8 to 14. The weight and stool changes of the mice were recorded daily, and the disease activity index (DAI) was established (Table 1) [18]. At the end of the experiment, blood was taken from the retro-orbital sinus, and the serum was obtained and centrifuged at 8000 rpm at 4 °C for 20 min, and stored at −80 °C. Then, the mice colonic samples were collected for further analysis.

### 2.5. Histopathology

The colon specimens were fixed in 10% formalin for 24 h and embedded in paraffin. After the paraffin-embedded sections were dewaxed and stained with haematoxylin and eosin, the degree of inflammation, depth of the lesion, crypt, and epithelial damage of the colon tissue were observed under a light microscope.

### 2.6. Determination of DAO, SOD, GSH-Px, and Inflammatory Cytokine Concentrations

The concentrations of diamine oxidase (DAO), IL-1β, IL-6, IL-10, and TNF-α in serum were determined with mouse ELISA kits (Shanghai Jianglai Biotechnology Co., Ltd., Shanghai, China) following the manufacturer’s instructions. The concentrations of superoxide dismutase (SOD) and glutathione peroxidase (GSH-Px) of the colon tissue were assessed with commercial kits (Jianglai, Shanghai, China).

### 2.7. 16S rRNA High Throughput Sequencing

Genomic DNA was obtained from frozen fecal samples using a DNA isolation kit (Jianglai, Shanghai, China). The structural changes of the intestinal microbiota were conducted by 16S rRNA sequencing analysis. The data were analyzed on the online platform Majorbio Cloud Platform (Majorbio, Shanghai, China).

### 2.8. Statistical Analysis

The results were represented as mean ± standard deviation (SD). DAI was assessed with the unpaired two-tailed Student’s *t*-test. Multiple comparisons were evaluated using one-way ANOVA followed by Tukey’s multiple-comparison test. *p* < 0.05 was considered significant.

## 3. Results

### 3.1. Preparation of APH

The preparation of bioactive peptides by enzymatic hydrolysis is widely used due to its high safety, mild reaction conditions and easy control of the hydrolysis process. In order to obtain corn protein hydrolysate with glutamine-rich peptides, Alcalase (A) and Protamex (P) were used to hydrolyze the CGM in this study, and the effect of a different order of enzyme addition (A + P and P + A) on the Gln content in the hydrolysate was compared. The content of Gln in the hydrolysate by A + P and P + A were 12.53% and 9.99%, respectively, showing a significant difference (*p* < 0.05). The sequential hydrolysis of A + P was more conducive to the release of glutamine peptides from CGM than P + A. Thus, the sequential hydrolysis of CGM by A + P was selected to prepare the APH.

### 3.2. APH Alleviates Colitis Symptoms in Mice

**Figure 1:**
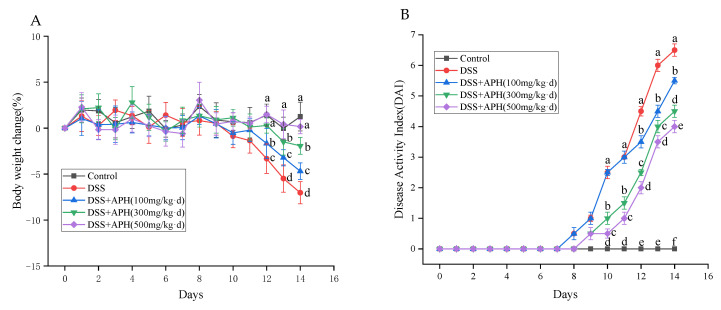

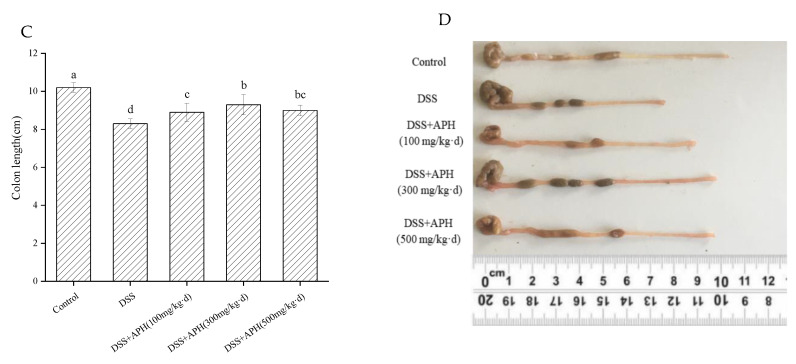
APH ameliorates clinical signs of colitis in mice. (**A**) Changes in body weight; (**B**) DAI scores; (**C**,**D**) colon length. Data are expressed as mean ± SD (*n* = 8). Different letters indicate significant differences (*p* < 0.05).

It is well known that the rate of change in body weight and DAI score are the main evaluation indexes of UC. The body weight change rate of all groups of mice before DSS treatment was low, and their activity status was normal (Figure 1A,B). DSS treatment was started on the eighth day: the activity of the mice decreased and the DAI score increased gradually with the increase of treatment time in the model group. As soon as the mice were treated with DSS for 4 days, they developed loose, sticky, and bloody stools. According to the above phenomena, the model could be considered successful. Compared with the DSS group, the rate of change in body weight and the degree of diarrhea and bloody stools in the APH groups were significantly improved (*p* < 0.05), especially in the APH (500 mg/kg·d) group.

The colon length of the animals usually shortens under inflammatory stress. The colon length was measured in each group (Figure 1C,D) and, compared to the control group, the model group’s colon length significantly shortened. The length of the colon of mice in the APH groups was considerably longer than those in the model group, especially in the medium-dose APH (300 mg/kg·d) group. This indicated that APH inhibited the intestinal stress response and alleviated colitis symptoms.

### 3.3. APH Alleviates Histopathological Changes in Mice

**Figure 2:**
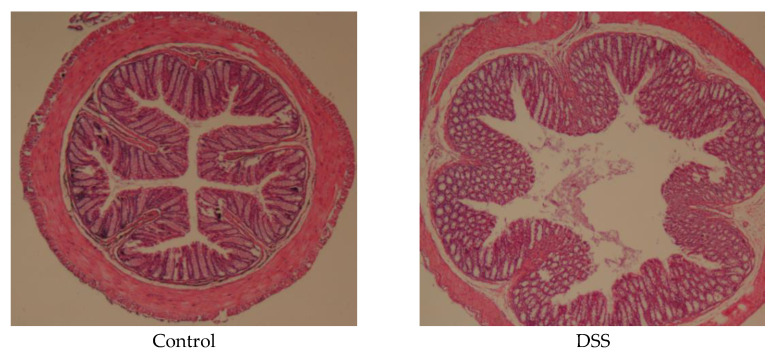

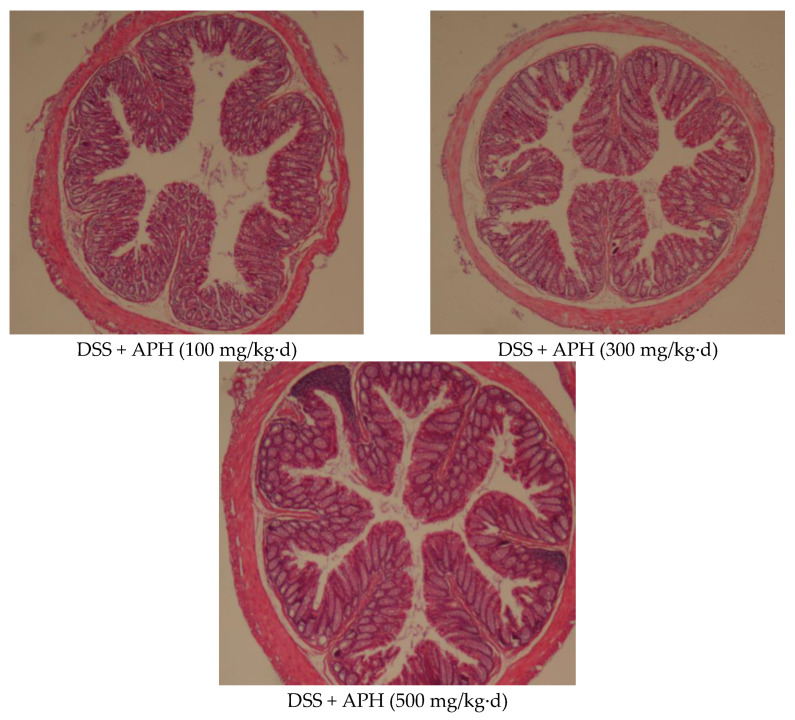
APH alleviates DSS-induced histopathological changes.

A pathologic examination was conducted to observe the extent of colonic injury (Figure 2). In the control group, the colonic mucosal layer was intact, there was a neat arrangement of glands, and no inflammatory cell infiltration was observed. The DSS group showed serious damage to the colonic mucosa, including edema, loss of some glands and crypt, and the infiltration of inflammatory cells. However, in the APH groups, the degree of inflammatory cell infiltration and colonic mucosa injury were reduced, especially in the high-dose APH group, where the structure of the colonic epithelial was more complete, and the colonic tissue injury was significantly improved. The colonic tissue morphology was similar to the control group, indicating that APH can substantially reduce the damage of colon tissue, and has a specific protective effect on the colon.

### 3.4. Effects of APH on the Inflammatory Cytokines in Mice

**Figure 3:**
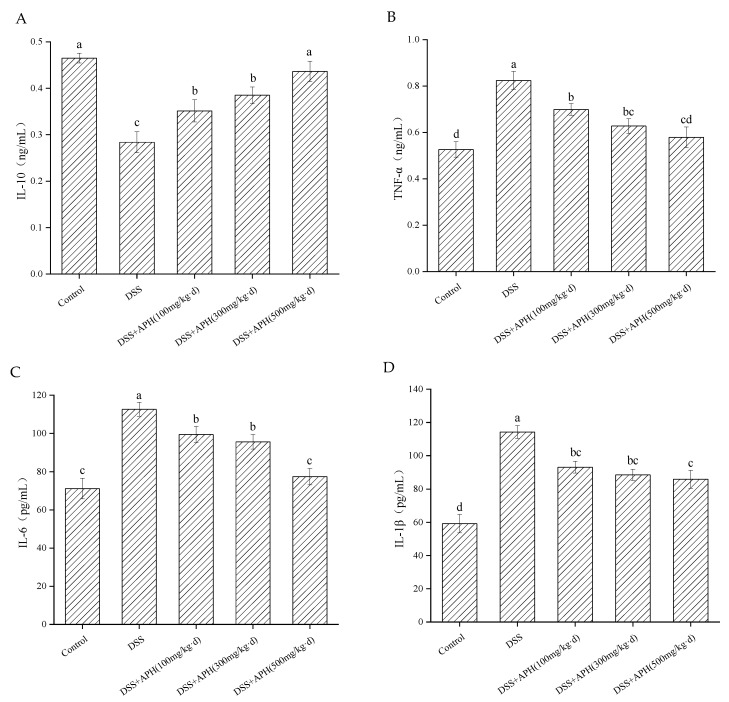
Effects of APH on the inflammatory cytokines, including the serum levels of IL-10 (**A**), TNF-α (**B**), IL-6 (**C**) and IL-1β (**D**). Data are expressed as mean ± SD (*n* = 8). Different letters indicate significant differences (*p* < 0.05).

Inflammatory cytokines are critical signaling molecules of inflammatory response in vivo, and colitis severity is closely related to their expression levels. The contents of inflammatory cytokines in serum were detected by ELISA (Figure 3). The serum IL-10 levels of mice in the DSS group were significantly lower than the control group (*p* < 0.05) (Figure 3A). In contrast, the levels of TNF-α, IL-6, and IL-1β were increased considerably (Figure 3B–D), indicating that colon inflammation was induced in mice after DSS treatment. After treatment with different doses of APH, the contents of IL-10 in the serum increased, and the contents of TNF-α, IL-6, and IL-1β decreased. These results indicated that APH played a specific role in regulating the levels of inflammatory factors, and it further revealed that APH had a particular anti-inflammatory effect on colitis in vivo.

### 3.5. Effects of APH on Oxidative Stress-Related Indicators in Mice

**Figure 4:**
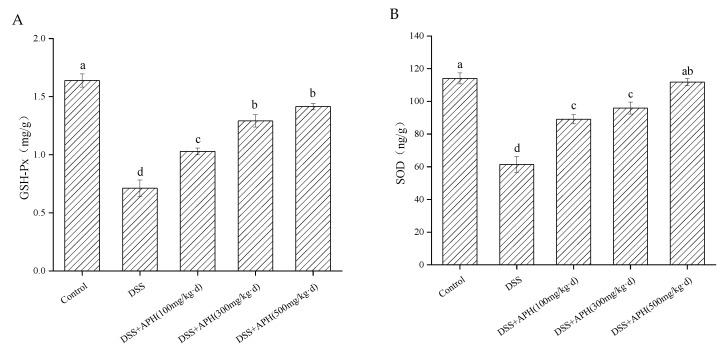
Effects of APH on the oxidative stress-related indicators, including the colonic concentrations of GSH-Px (**A**) and SOD (**B**). Data are expressed as mean ± SD (*n* = 8). Different letters indicate significant differences (*p* < 0.05).

To further evaluate the effect of APH on UC from the perspective of oxidative stress, two oxidative stress markers, GSH-Px and SOD in the colon tissue, were examined (Figure 4). The contents of GSH-Px and SOD in the model group were significantly lower than in the control group (*p* < 0.05). The levels of the two oxidative stress markers were significantly increased in a dose-dependent manner after treatment with different doses of APH, indicating that APH could improve the antioxidant capacity of mice with colitis, and had a specific regulatory effect on oxidative stress.

### 3.6. Effects of APH on Intestinal Permeability in Mice

**Figure 5:**
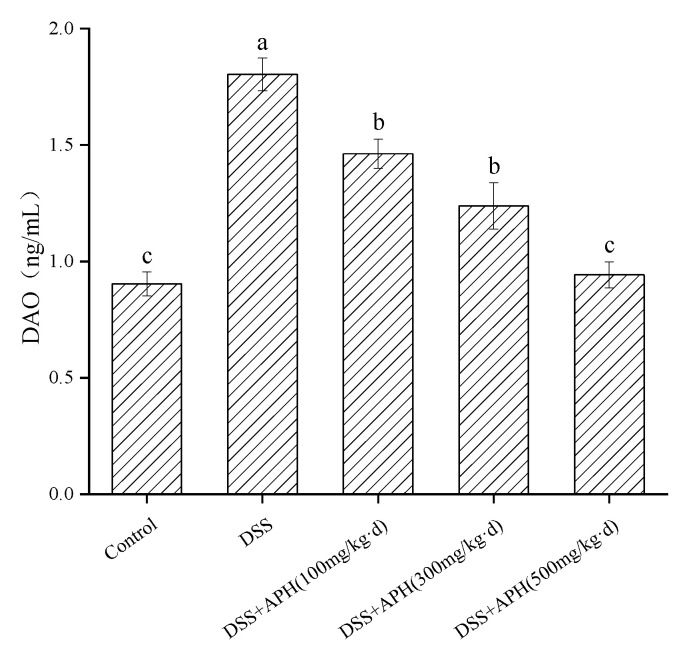
Effects of APH on DAO level in serum of DSS-induced colitis mice. Data are expressed as mean ± SD (*n* = 8). Different letters indicate significant differences (*p* < 0.05).

Diamine oxidase (DAO) is an intracellular enzyme with strong activity located in the upper layer of the intestinal villi in mammals. It is also a marker enzyme of intestinal mucosal cells. When intestinal mucosal epithelial cells are damaged, the intracellular DAO releases into the intestine and blood, which increases the level of DAO in the blood, indicating the increase of intestinal permeability [19]. Therefore, the change of DAO level is closely related to the integrity and damage degree of the intestinal mucosal mechanical barrier, and can be used as a sensitive indicator to reflect the change of intestinal permeability. The serum DAO levels in the DSS group were significantly higher (*p* < 0.05) than in the control group (Figure 5), indicating that the intestinal mucosa of the mice in the model group was severely damaged and the permeability increased. After APH treatment with different doses, the serum DAO levels of the mice significantly decreased (*p* < 0.05), especially in the high-dose APH group. These results indicated that APH could reduce the damage to the intestinal mucosa, reduce the permeability of the intestinal mucosa, and exhibit a particular protective effect on the mechanical barrier of the intestinal mucosa.

### 3.7. Effects of APH on Intestinal Microbiota in Mice

**Figure 6:**
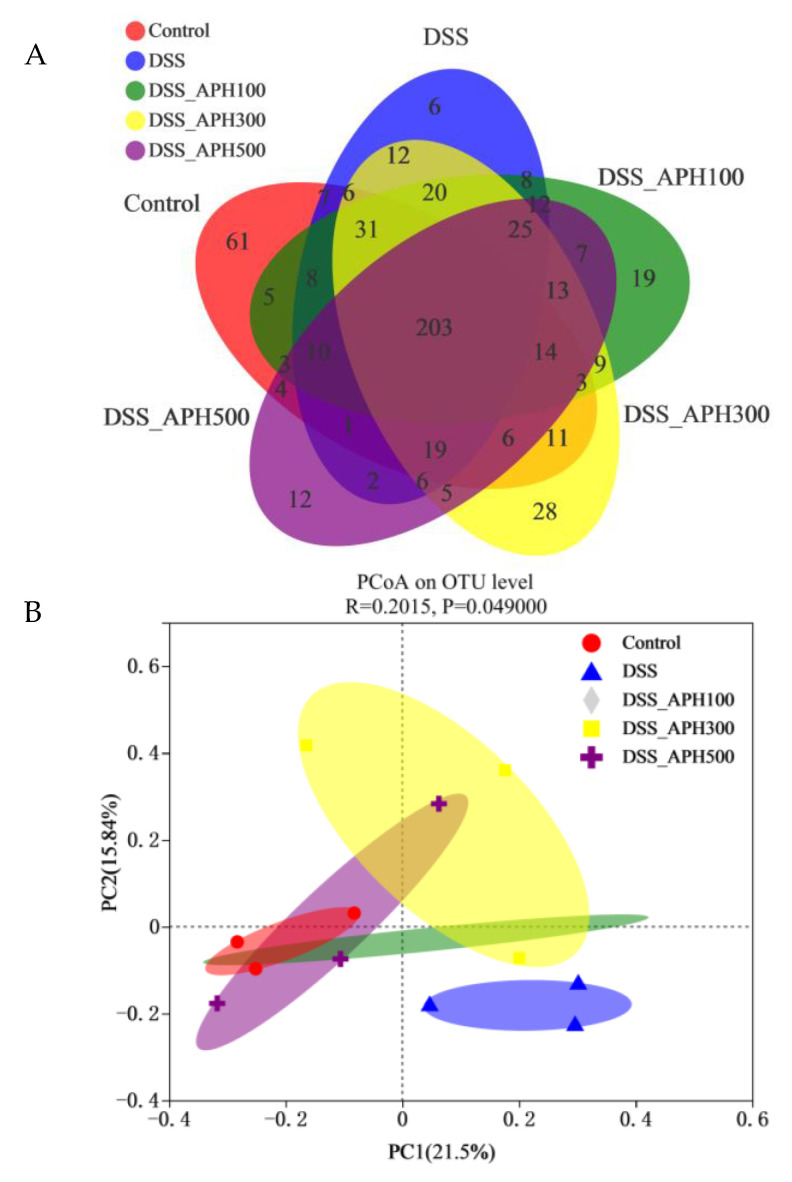

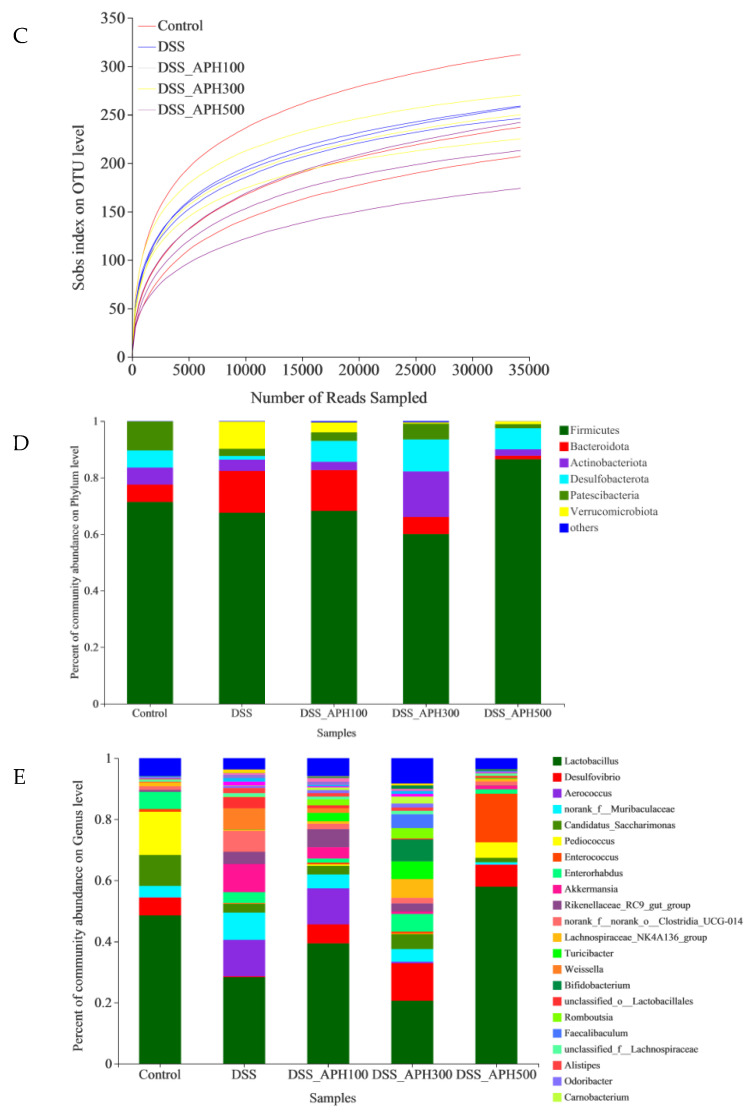
Effects of APH on the intestinal microbiota in mice with DSS-induced colitis. (**A**) Venn diagrams of OTU of each group; (**B**) principal coordinate analysis (PCoA); (**C**) rarefaction curve; (**D**) microbiota analysis at the phylum level; (**E**) microbiota analysis at the genus level.

The intestinal microbiota of mice were measured using 16S rRNA high-throughput sequencing. A total of 203 OTUs were shared by the five groups, and the unique OTUs of each group was 61, 6, 19, 28, and 12, respectively (Figure 6A). This indicated that DSS reduced the diversity of the intestinal microbiota in mice, and the number of OTUs increased after APH treatment with different doses. Principal coordinate analysis (PCoA) can reflect the similarity between the samples: the shorter the distance between the sample and the sample in the figure, the more similar the samples are. The control group was closely clustered (Figure 6B), and there was an obvious dividing line and a long distance with the DSS group, which indicated that DSS had a particular influence on the intestinal microbiota. After APH treatment with different concentrations, the abundance and structure of the intestinal microbiota of the mice improved. The results of alpha diversity analysis demonstrated that DSS reduced the diversity and richness of the intestinal microbiota (Figure 6C), while APH improved it.

The analysis results of intestinal microbiota abundance at the phylum level showed that the overall microbiota composition predominantly changed in the DSS group: the relative abundance of *Firmicutes*, *Actinobacteriota*, *Desulfobacterota*, and *Patescibacteria* significantly decreased, while *Bacteroidota* and *Verrucomicrobiota* increased (Figure 6D). After treatment with different concentrations of APH, the relative abundance of Firmicutes in the high-dose APH (500 mg/kg·d) group and *Actinobacteriota*, *Desulfobacterota*, and *Patescibacteria* in the medium-dose APH (300 mg/kg·d) group significantly increased, while the relative abundance of *Bacteroidota* and *Verrucomicrobiota* significantly decreased.

The intestinal microbiota also changed significantly at the genus level (Figure 6E). The abundance of Lactobacillus, a family that regulates intestinal health, decreased in the DSS group. The abundance of other genera including *Desulfovibrio* and *Pediococcus* in the DSS group also decreased. After treatment with different concentrations of APH, the relative abundance of *Lactobacillus* and *Pediococcus* in the high-dose APH (500 mg/kg·d) group and *Desulfovibrio* in the medium-dose APH (300 mg/kg·d) group significantly increased, while the relative abundance of *Aerococcus* significantly decreased.

## 4. Discussion

As a high-quality protein raw material rich in Gln, CGM was hydrolyzed by two commercial microbial proteases to prepare APH in the present study. Alcalase is produced by the fermentation of *Bacillus licheniformis*, which is an endopeptidase with serine in its catalytic site. Protamex, produced from *Bacillus subtilis*, is also an endopeptidase with broad specificity for peptide bonds. It has been reported that Alcalase and Protamex have little effect on the hydrolysis of the amide group in Gln, and can effectively protect the Gln in the raw material [20,21]. According to the results of this study, the content of Gln in the hydrolysate prepared by the sequential hydrolysis of A + P was significantly higher than that of P + A. It is possible that the cleavage sites of the two proteases are different, and the change of enzyme addition order will lead to different hydrolysates, which will result in the difference of Gln content in the hydrolysates. During the first step of hydrolysis, Alcalase showed a more intense cleavage effect on CGM than Protamex, which resulted in a relatively high Gln content in the hydrolysate. More importantly, more sites were exposed in CGM after the hydrolysis of Alcalase, which was beneficial for Protamex to play a better role in the second step of hydrolysis. On the contrary, the cleavage site of Alcalase was preempted if the Protamex was used as the first hydrolase, which is the reason why Alcalase exhibited lower hydrolysis effects on the residual peptides after the action of Protamex, and the Gln content in the hydrolysate was lower than that of A + P. Thus, the sequential hydrolysis of CGM by A + P was more suitable for the preparation of the APH.

Intestinal mucosal barrier injury is a typical symptom experienced by UC patients, and the pathological manifestation is increased intestinal permeability. With the increase of intestinal permeability, harmful substances such as bacteria and endotoxins can enter the body and affect human health. Preventing mucosal damage or promoting the efficient regeneration of mucosal cells is one of the ways to alleviate UC. As an essential amino acid of the intestinal tract, Gln is the main energy source of the intestinal cells that contribute to enhancing the metabolism and synthesis of mucosal proteins and cells, promote the regeneration and renewal of intestinal mucosal cells, and effectively protect the integrity of cells [22]. The corn protein hydrolysate prepared in this study was rich in Gln peptides, which significantly alleviated colonic tissue damage and reduced the intestinal permeability in mice with colitis. The effect of APH in the high-dose group was significantly better than that in the low-dose group. It could be speculated that Gln peptides, especially Gln, might play an important role in antagonizing UC. Evidence shows that under severe stress such as infection or trauma, the Gln in intestinal mucosal epithelial cells is rapidly depleted. The timely supplementation of Gln can effectively prevent intestinal mucosal atrophy [23,24]. Further investigation is required to confirm the mechanism of glutamine peptide in APH.

The infiltration of neutrophils in intestinal mucosa and epithelial cells is another feature of UC, which can impair epithelial barrier function, damage tissues, release pro-inflammatory factors, and increase inflammation. Studies have shown that inflammatory mediators have an important relationship with inflammatory bowel disease. The excessive production of pro-inflammatory factors will intensify the inflammatory cascade and cause colon injury, forming a vicious cycle [25]. IL-6 is a crucial mediator in UC inflammation produced by T cells and macrophages [26]. TNF-α, the earliest endogenous mediator of inflammatory processes in tumors, can promote the diffusion and differentiation of T cells, disrupting the intestinal barrier [27]. IL-1β is involved in the recruitment and retention of leucocytes in inflammatory tissues [28], while IL-10 is an anti-inflammatory factor involved in immune regulation and produced by regulatory T cells and mononuclear macrophages. IL-10 can inhibit the development of UC by downregulating inflammatory signaling and mucosal inflammation. The dynamic balance between inflammatory cytokines determines the development and outcome of inflammation. The present study found that APH reduced the inflammatory response by upregulating the content of IL-10 and downregulating the content of TNF-α, IL-1β, and IL-6, which demonstrates that APH could alleviate ulcerative colitis. The underlying regulatory mechanism may be related to Gln peptide or Gln in APH. Gln is an important fuel for the immune system: it plays a crucial part in immune regulation and can enhance the function of the immune system [29]. The proliferation and activation of immune cells can effectively regulate the synthesis and secretion of inflammatory cytokines, leading to the regulation of immune homeostasis. Furthermore, NF-κB is one of the main signaling pathways mediating the inflammatory response. It is mediated by Toll-like receptor 4 and induces intracellular signal transduction through myeloid differentiation factor 88, which activates NF-κB and leads to the increased secretion of inflammatory cytokines. It has been found that Gln supplementation can regulate the activation of NF-κB to inhibit inflammation in colitis models [30]. Mengya Zhang et al. also reached the same conclusion when studying the preserved egg-white anti-inflammatory peptides [31]. Meanwhile, the function of hydrolysate is closely related to the amino acid sequence of peptides in the hydrolysate. Peptides with anti-inflammatory activity were rich in positively-charged and hydrophobic amino acids [32]. It has been reported that one or more residues of Gln, Pro, Tyr, Trp, Cys, Ala, and Asp might contribute to the immunomodulatory activity of peptides [33]. In the present study, CGM, the raw material for the preparation of APH, contains lots of Gln and hydrophobic amino acids (Table S1). The hydrophobic amino acid content accounts for about 50% of the total amino acid content. Therefore, many peptides containing hydrophobic amino acids may be obtained after the enzymatic hydrolysis of CGM. Hence, we speculate that the antagonistic effect of APH on UC might be related to the special amino acid sequence in peptides. González-Montoya et al. reported that the anti-inflammatory peptides obtained from germinated soybean protein hydrolysates all contained Gln residue, and the identified peptides were rich in Gln, which had strong anti-inflammatory activity, such as QQQQQGGSQSQ, QEPQESQQ, QQQQQGGSQSQSQKG, and PETMQQQQQQ [34]. It further confirmed our speculation, and more research is necessary to elucidate the mechanics of Gln and hydrophobic amino acids in antagonizing UC.

The inflammatory response is closely related to oxidative stress. Oxygen radicals are the initiator of inflammatory response, which can accelerate the release of inflammatory factors and aggravate the damage of the inflammatory response [35]. Oxidative stress not only produces inflammation in the gut, but also causes tissue damage. Studies have shown that the overproduction of reactive oxygen and nitrogen, as well as their associated oxidative stress regulation, are related to UC [36]. Yuji Naito et al. reported that ROS levels in the colon samples of UC patients significantly increased and were positively correlated with IBD [37]. Two primary markers (GSH-Px and SOD) of oxidative stress were detected in our study, and the results revealed that APH increased the levels of GSH-Px and SOD. As a precursor of glutathione, Gln supplementation can effectively maintain and increase the storage of glutathione in cells and improve the body’s antioxidant capacity, thereby stabilizing the cell membrane and protein structure, and maintaining the function of intestinal and immune cells. This might be a major reason why APH significantly regulates GSH-Px and SOD levels. On the other hand, corn protein hydrolysate contains lots of hydrophobic amino acids, which contribute to its antioxidant activity and may also increase anti-inflammatory activity [38]. Our previous studies have also shown that CGM hydrolysates contain substances that activate SOD and GSH-Px factors, which interact with free radicals to produce stable products [16]. Therefore, APH possibly played a positive role in relieving oxidative stress and subsequently reducing tissue damage. The imbalance between tissue oxidation and antioxidant reaction is the basis of disease progression in many cases, so APH can also be used from an antioxidant perspective to reduce the severity of UC.

The stability of intestinal microecology is the basis of intestinal health, which is directly related to the structure and function of the intestinal barrier [39]. The intestinal microbiota are a large number of microorganisms colonized in the human digestive tract, with diverse functions and complex species. Different species in the normal intestinal microbiota, as well as the intestinal microbiota and the host, are always in a state of dynamic balance, forming an interdependent and mutually restricted system. The intestinal microbiota are beneficial to the human body under normal circumstances. However, when the system is out of balance, it might cause or exacerbate chronic inflammation. In the present investigation, the alpha-diversity analysis, including the rarefaction curve, indicated that APH at different concentrations could effectively prevent the change of the diversity and abundance of intestinal microbiota. According to the beta-diversity analysis, DSS caused intestinal microflora disorder in mice, while APH could effectively alleviate this phenomenon. Recent research also showed that the structure of the intestinal microbiota plays a crucial role in UC [40]. Haolong Zhang et al. reported that the proliferation of probiotics such as lactobacillus can alleviate the severity of UC [41]. Xiaokun Cai et al. found that increasing beneficial bacteria and reducing harmful bacteria could partially reverse the changes in intestinal microbiota, thus alleviating the symptoms of colitis [42]. Up to now, intestinal microflora have become a new target of gastrointestinal disease treatment, while APH could improve the symptoms of colitis by regulating the structure and abundance of intestinal microflora. Therefore, APH can be further studied as a functional food to prevent UC.

Accumulating evidence has shown that in patients with UC, there are fewer probiotics and more pathogenic and harmful bacteria [43]. Powell and Chen reported that *Bacteroides* and *Proteobacteria* can promote the intestinal tract to produce excessive pro-inflammatory cytokines, and they are the main pathogenic bacteria of colitis [44,45]. As the major metabolites of probiotics, short-chain fatty acids (SCFAs) have been shown to increase tight junction protein expression and reduce pro-inflammatory cytokine production in intestinal epithelial cells [46]. At the phylum and genus levels in this study, we found that the relative abundance of *Bacteroides* and *Aerococcus* was enhanced by DSS, yet suppressed with APH treatment. In addition, APH promoted the abundance of probiotics such as *Lactobacillus*. This presumably might be due to the fact that APH increased the content of SCFAs by regulating the abundance of probiotics, thereby reducing the level of pro-inflammatory factors and alleviating the symptoms of colitis. Moreover, the decreased level of pro-inflammatory cytokines in the APH groups might be related to the reduced abundance of *Bacteroidetes*. This further indicated that regulating intestinal microbiota may be one of the mechanisms by which APH exerts anti-UC effects.

In conclusion, the results obtained in this study clearly demonstrate that APH could effectively relieve DSS-induced ulcerative colitis in vivo, which played its role mainly by regulating the level of inflammatory cytokines and the structure of intestinal microbiota. Overall, our study provided a new strategy for antagonizing UC, and also offered a high-value utilization for CGM.

## Figures and Tables

**Table 1:** Disease activity index scoring system.

Score	Weight Loss (%)	Stool Consistency	Occult/Gross Bleeding
0	0	Normal	Normal
1	1–5	-	-
2	6–10	Loose	Hemoccult positive
3	11–15	-	-
4	>16	Diarrhea	Gross bleeding

## Data Availability

Data is contained within the article or Supplementary Material.

## Supplementary Materials

The following supporting information can be downloaded at: www.mdpi.com/article/10.3390/foods11213359/s1, Table S1. Amino acid composition of CGM and APH.
