# Supplementary material for: Production of Corn Protein Hydrolysate with Glutamine-Rich Peptides and Its Antagonistic Function in Ulcerative Colitis In Vivo
Source: Foods. 2022 Oct 25;11(21):3359. doi: 10.3390/foods11213359 (PMC9657542; doi:10.3390/foods11213359)
Supplement: Supplementary file 1 [file foods-11-03359-s001.zip › foods-1948685-supplementary.pdf]

Supplementary Material

Supplementary Material 1:

Table S1. Amino acid composition of CGM and APH

Amino Acid #	CGM (g 100 g^{-1})	APH (g 100 g^{-1})
Asx	3.35	4.80
Thr	1.97	2.92
Ser	3.08	4.38
Glx	12.55	16.81
Gly	1.53	2.07
Ala	4.91	6.65
Cys	0.31	0.00
Val	2.36	2.66
Met	1.18	0.84
Ile	2.08	2.25
Leu	9.88	12.15
Tyr	2.99	4.15
Phe	3.52	4.43
Lys	0.92	1.11
His	1.18	1.48
Arg	1.80	2.09
Pro	8.03	10.31
THA*	31.24	39.29
TAA*	61.63	79.10

#Asx represents Asn and Asp; Glx represents Gln and Glu.

*THA represents Total hydrophobic amino acid; TAA represents Total amino acid.
